# Calx, a sodium/calcium exchanger, may affect lifespan in *Drosophila melanogaster*

**DOI:** 10.17912/micropub.biology.000220

**Published:** 2020-02-12

**Authors:** Jung-Wan Mok, Hyunglok Chung, Kwang-Wook Choi

**Affiliations:** 1 Department of Biological Sciences, Korea Advanced Institute of Science and Technology, Daejeon 34141, Korea; 2 Current address: Department of Molecular and Human Genetics, Baylor College of Medicine, Houston, TX 77030, USA

**Figure 1:**
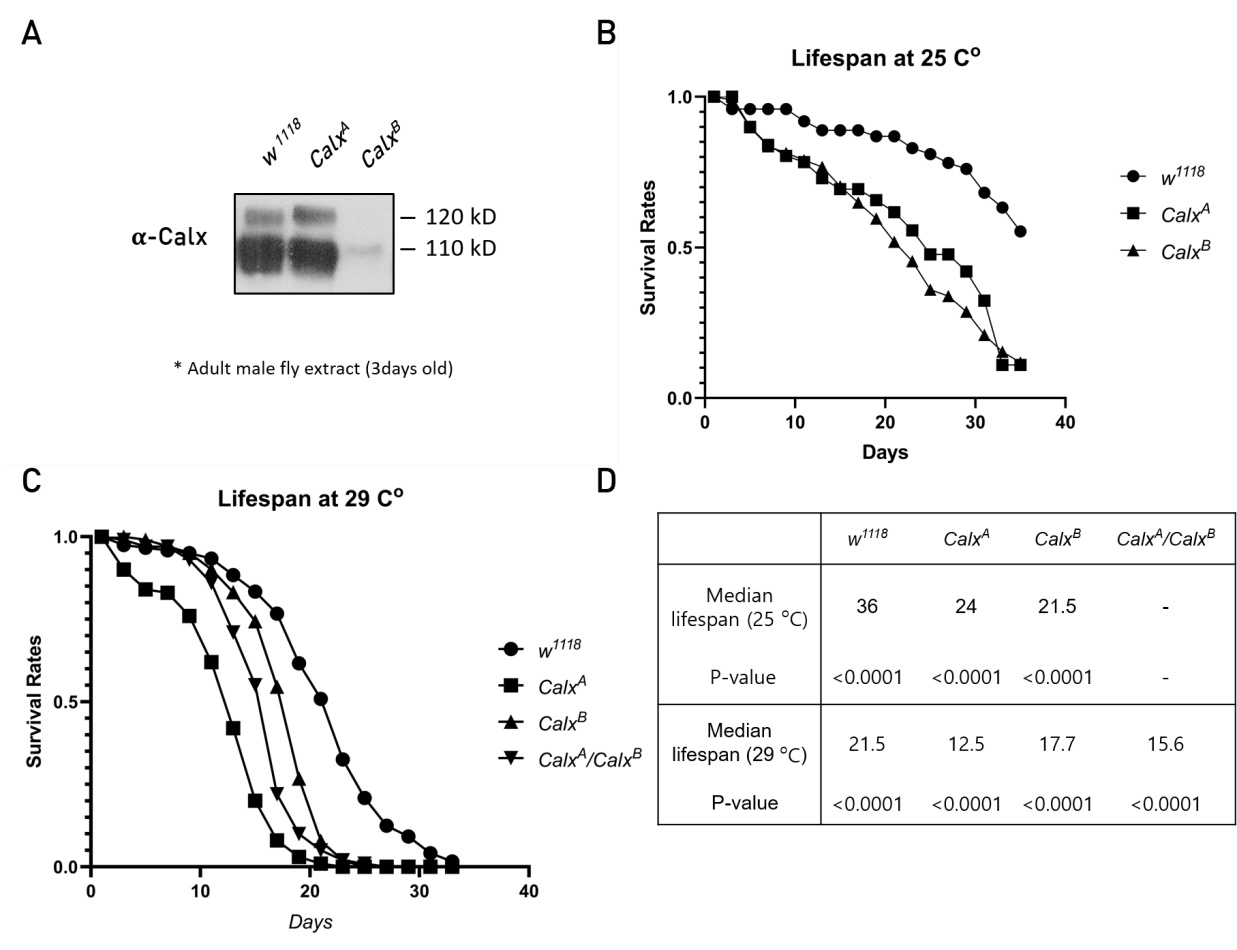
A. Calx protein expression profile in *Calx* homozygous mutants. B. Lifespan assay at 25℃. C. Lifespan assay at 29℃. D. Statistics for (B) and (C).

## Description

Calcium homeostasis is essential for normal body function.Calx is a *Drosophila* homolog of the mammalian sodium/calcium exchanger (NCX) involved in the regulation of intracellular calcium (Ca^2+^) level (Schwarz and Benzer 1997). A major function of Calx is to export excess Ca^2+^ to the outside of the cell when intracellular Ca^2+^ level is elevated (Hryshko **et al.** 1996). Genetic studies in *Drosophila* have shown that *Calx* mutants can develop to adult flies, even though Calx is expressed throughout the developmental stages (Wang **et al.** 2005). However, Ca^2+^ efflux mediated by Calx is crucial for maintaining the cellular Ca^2+^ homeostasis in light-activated sensory neurons (Wu **et al.** 2011). In addition to its role for phototransduction, Calx is also required for preventing light-induced retinal degeneration caused by Ca^2+^ overload (Wang **et al.** 2005).

Thus far, Calx function has been extensively studied in the retinal cells. However, expression of *Calx* gene is not restricted to the eye, and potential functions of Calx in other tissues are largely unknown. Interestingly, disrupted Ca^2+^ homeostasis has been implicated in aging and is regarded as one of the biomarkers of aging (Foster and Kumar 2002). Although mammalian NCXs have been implicated in aging of tissues and organs (Gomez-Villafuertes **et al.** 2007; Zhang **et al.** 2014), it remains to be studied whether NCXs are important for animal lifespan. Here, we address the question whether Calx affects lifespan in fly.

To determine whether Calx is required for normal lifespan, we carried out genetic analysis using two *Calx* mutants, *Calx^A^* and *Calx^B^*. It has been shown that homozygous *Calx^A^* and *Calx^B^* mutant flies are viable (Wang **et al.** 2005). *Calx^A^* mutants have a point mutation (T822I) but show relatively normal level of protein expression in adult head. In contrast, *Calx^B^* is a regulatory mutation that causes reduced protein expression (Wang **et al.** 2005). To check whether such mutant effects can also be seen in non-head tissues, we examined Calx protein expression in whole adult fly body. It has been reported that Calx is expressed as a single 110 kD protein in adult head and body (Wang *et al.*, 2005). *w^1118^* flies used as wild-type control showed a major band at 110 kD and a weaker band at 120 kD (Fig. 1A). Consistent with earlier studies (Wang **et al.** 2005), we found a strong reduction in the 110 kD band in *Calx^B^* mutant, while *Calx^A^* mutant showed a nearly indistinguishable pattern from wild-type. *Calx^B^* mutant showed severe reduction in both 110 and 120 kD bands, suggesting that Calx exists at least in two different forms in adult fly.

Next, we measured lifespan of wild-type and *Calx* males. We used isogenic *w^1118^* as control since it was previously used as control for analyzing *Calx* mutant phenotypes in phototransduction (Wang **et al.** 2005). In our lifespan assay at 25 ^o^C, both *Calx^A^* and *Calx^B^* mutants showed considerable reduction in the survival rate compared with the wild-type control (Fig. 1B). Median lifespan of *Calx^A^* and *Calx^B^* mutants was 24 and 21.5 days, respectively, whereas the median for wild-type was about 36 days (Fig. 1D). We also examined survival rates of *Calx* mutants at 29 ^o^C (Fig. 1C). At this high temperature, wild-type flies showed a lower survival rate with median lifespan of 21.5 days. Under this condition, *Calx^A^* mutant flies had a significantly shorter median lifespan than *Calx^B^* mutant (12.5 days for *Calx^A^;* 17.7 days for *Calx^B^*) (Fig. 1D). Since *Calx^A^* encodes a mutated protein, it is possible that the mutant Calx protein might be more unstable at 29 ^o^C, causing more severe phenotype in longevity. Alternatively, *Calx^A^* mutantcells may require a higher rate of calcium extrusion at the upper extreme of their viable temperature range than their more normal growth temperature (25 ^o^C). We also checked the lifespan of transheterozygotes for *Calx^A^* and *Calx^B^* mutations (*Calx^A^/Calx^B^)*. Trans-heterozygous flies also showed significantly reduced lifespan compared with wild-type, indicatingthat *Calx^A^* and *Calx^B^* fail to complement the longevity phenotype. Consistent with the hypomorphic nature of the *Calx^B^* allele, trans-heterozygous flies showed an intermediate lifespan between *Calx^A^* and *Calx^B^* mutants (Fig. 1C, D). Taken together, our data suggest that the *Calx* mutations are responsible for the observed phenotype of shorter longevity. These results are preliminary, and to rule out potential genetic background effects, additional controls should be included in the future.

This study raises a possibility that Calx might be required for normal lifespan. The Ca^2+^ influx channel TRP, which has an opposite function to Calx, is crucial for phototransduction (Montell 2005; Wang **et al.** 2005). The *trp* gene has a paralog, *trp-like* (*trpl*) (Phillips **et al.** 1992). Although there is no strict paralog of *Calx* in *Drosophila*, *Nckx30C* is a distant relative of *Calx* (Haug-Collet **et al.** 1999). It remains to be tested whether these related genes are also required for normal lifespan. Importantly, retinal degeneration by loss of TRP or constitutive TRP function can be suppressed by defects or overexpression of Calx, respectively. Further studies are necessary to see whether lifespan is dependent on the functional relationship between Calx and TRP in the intracellular Ca^2+^ regulation, although such genetic analysis may be complicated by the existence of related genes like *trpl* and *Nckx30C*. It is also an intriguing question whether NCX homologs of Calx play a role in lifespan of mammals.

## Methods

*Western Blot –* For western blot experiment, 10 adult male flies for each genotype (3 days old virgin) were used for protein extraction in 200μl of SDS sample buffer. After boiling for 5 minutes at 94 °C, samples were centrifuged at 12000g for 10 minutes. Supernatants were loaded on 10% polyacrylamide gel. Fractionated proteins were transferred by western blotting for immunostaining (Calx antibody 1:1000, kind gift from Dr. Craig Montell).

*Lifespan assay* – For lifespan assay, one hundred adult male flies of each genotype (1 day old, virgin) were collected by minimal exposure to carbon dioxide gas. Flies were raised in food vials (10 males/vial) and transferred to new vials every 2-3 days. Number of dead flies was recorded every day. Statistical significance of the data from lifespan assay was determined by P-value evaluation from two-tailed T test.



## Reagents

*Fly strains* – Isogenized *w^1118^* (#5905), *Calx^A^* (#24496) and *Calx^B^* (#24497) mutants were kindly provided by the Bloomington Drosophila Stock Center.
